# DeepLabCut-based automated system reveals diverse temperature tolerance among medaka strains and related *Oryzias* species

**DOI:** 10.1038/s41598-026-66712-w

**Published:** 2026-09-10

**Authors:** Yoshiya Matsuo, Takuya Kato, Kiyoshi Naruse, Takashi Yoshimura, Tatsuhito Hasegawa, Tomoya Nakayama

**Affiliations:** 1 Laboratory of Animal Integrative Physiology, Graduate School of Bioagricultural Sciences, Nagoya University, Nagoya, 464-8601 Japan; 2 Graduate School of Engineering, University of Fukui, Fukui, 910-8507 Japan; 3 Department of Basic Biology, School of Life Science, SOKENDAI (The Graduate University for Advanced Studies), Okazaki, 444-8787 Japan; 4 Laboratory of Bioresources, National Institute for Basic Biology, Okazaki, 444-8585 Japan; 5 Institute of Transformative Bio-Molecules (WPI-ITbM), Nagoya University, Nagoya, 464-8601 Japan; 6 Center for One Medicine Innovative Translational Research (COMIT), Nagoya University, Nagoya, 464-8601 Japan; 7 Private company, Tokyo, Japan

**Keywords:** Thermal tolerance, Loss of equilibrium, Automated phenotyping, DeepLabCut, Medaka, Computational biology and bioinformatics, Ecology, Ecology, Genetics, Zoology

## Abstract

Temperature is a critical environmental factor influencing the physiology and behavior of ectothermic animals, yet conventional methods for evaluating thermal tolerance in fish rely on subjective manual observation of loss of equilibrium (LOE), limiting experimental throughput and introducing observer bias. Here, we developed an automated temperature tolerance evaluation system integrating DeepLabCut-based pose estimation with custom image processing algorithms to objectively quantify the timing of LOE during thermal stress tests. Our system incorporated region partitioning and color transformation preprocessing to improve keypoint detection accuracy, followed by a classification model combining ResNet34-based frame features with keypoint coordinates to objectively determine the timing of LOE without manual observation. Validation against manual annotation showed that the automated system achieved an accuracy comparable to the natural variability between trained investigators, and outperformed naive human observers, supporting its validity as an objective and reproducible alternative to manual scoring. Using this system, we characterized cold and heat tolerance across six medaka strains (*Oryzias latipes*: d-rR/TOKYO, HB11A, OK-Cab, HO5 and HdrR-II1; *O. sakaizumii*: HNI-II). Cold and heat tolerance assessment revealed inter-strain variation, with HdrR-II1 among the most cold- and heat-tolerant strains and HNI-II the least tolerant of both cold and heat stress. We further evaluated cold tolerance in medaka-related species (*O. sinensis*, *O. cabaranensis*, *O. curvinotus*, *O. luzonensis*, *O. celebensis*, and *O. javanicus*) and zebrafish (*Danio rerio*), revealing substantial interspecific variation that broadly corresponded with latitudinal distribution. *O. latipes*, distributed at the highest latitudes among the tested species, exhibited the greatest cold tolerance, whereas *O. celebensis*, *O. javanicus*, and other tropical or low-latitude species showed comparatively low cold tolerance. Our automated system provides a robust, high-throughput platform for thermal tolerance evaluation and, combined with the genetic and genomic resources available in medaka, establishes a foundation for elucidating the molecular mechanisms underlying temperature adaptation in fish.

**Supplementary Information:**

The online version contains supplementary material available at https://doi.org/10.1038/s41598-026-66712-w.

## Introduction

Temperature is one of the environmental factors that have a strong influence on the physiological functions and behaviors of fish. Since fish are ectothermic animals that cannot control their body temperature, their internal biochemical reactions change dramatically with environmental temperature fluctuations. Throughout evolutionary history, fish species have developed specific thermal tolerance ranges that reflect their native habitat conditions, enabling optimal performance within species-specific temperature windows. However, anthropogenic climate change presents an unprecedented challenge to these evolved adaptations. Rising global temperatures have already begun to alter aquatic ecosystems worldwide, with water temperatures increasingly surpassing historical baselines^1–4^. Current projections from the Intergovernmental Panel on Climate Change indicate that, without a strengthening of policies beyond those implemented by the end of 2020, global warming of 3.2 [2.2 to 3.5]°C is projected by 2100^5^, which threatens to exceed the thermal tolerance limits of numerous fish species, potentially leading to population declines, range shifts, and local extinctions.

Characterizing interspecific and intraspecific variation in thermal tolerance is essential for predicting the ecological consequences of climate change on fish populations. Three principal methodologies have been established for quantifying thermal tolerance in fishes: the incipient lethal temperature (ILT) method, the chronic lethal method (CLM) and the critical thermal method (CTM)^6^. The ILT technique involves abruptly transferring groups of fish from their acclimation temperatures into a series of constant test temperatures near their estimated upper and lower temperature limits. Mortality is recorded over time, and the temperature tolerated by 50% of the sample for various exposure time intervals (e.g., 12, 24, 48, 96 h) is calculated. The CLM, in contrast, uses gradual temperature changes (1–2 °C per day) with mortality as the endpoint. The CTM has emerged as the most widely adopted approach, exposing fish to linear temperature changes (typically 0.3 °C per minute) until reaching a sublethal endpoint such as loss of equilibrium (LOE). The CTM offers several advantages: it is non-lethal when fish are promptly returned to acclimation temperatures, requires fewer test subjects than ILT protocols, and better approximates natural thermal regimes where temperature changes occur gradually rather than abruptly. However, conventional CTM implementations face significant limitations, including reliance on subjective visual observation to determine LOE, which introduces inter-observer variability and potential bias^7–9^. Furthermore, the labor-intensive nature of manual observation restricts experimental throughput and precludes detection of subtle individual-level or population-level differences in thermal tolerance.

Recent advances in machine learning and computer vision offer promising solutions to overcome these methodological limitations. DeepLabCut, a deep learning framework for markerless pose estimation, has demonstrated high accuracy in tracking animal behavior across diverse taxa^10–13^. However, several challenges remain when applying DeepLabCut to thermal tolerance assays. First, as a general-purpose tool, DeepLabCut cannot readily incorporate domain-specific prior knowledge, such as the physical structure of experimental arenas or phenotypic variation among individuals, which can limit detection accuracy under variable conditions. Second, its multi-animal tracking mode (maDLC) is prone to identity swaps when individuals are in close proximity and to labeling errors caused by environmental noise, resulting in higher keypoint detection errors compared with single-animal tracking. Third, DeepLabCut does not consider temporal continuity across frames, which can lead to sporadic large errors in individual frames. Finally, DeepLabCut is designed for keypoint detection and tracking, and does not inherently provide behavioral state classification such as the detection of LOE events. Addressing these challenges requires task-specific preprocessing, classification models, and post-processing steps tailored to the experimental context.

To develop and validate an automated thermal tolerance assessment system, we selected medaka (*Oryzias latipes* and *O. sakaizumii*) as our model organisms. Medaka presents several advantages for this application: (1) availability of numerous well-characterized inbred strains derived from populations inhabiting distinct thermal environments^14^ (2) the existence of closely related species within the genus *Oryzias* that are distributed across a wide range of thermal habitats from tropical to temperate regions, providing an interspecific comparative framework; (3) fully sequenced genome and established genetic resources facilitating future investigations of molecular mechanisms underlying thermal tolerance; and (4) small body size and short generation time enabling high-throughput experimental designs. The existence of genetically defined inbred strains originating from different latitudes, together with the rich species diversity within the genus, provides a powerful comparative framework for identifying genomic regions and physiological mechanisms associated with thermal tolerance variation.

In this study, we developed an integrated system that combines DeepLabCut-based keypoint detection with a ResNet-based image classification model to enable automated, objective detection of LOE timing during thermal stress tests. We applied this system to characterize thermal tolerance diversity across six medaka strains and six related *Oryzias* species, along with zebrafish (*Danio rerio*) as a widely used model fish species. Our automated approach eliminates observer bias, increases experimental throughput, and provides precise temporal resolution for detecting LOE events. This methodology not only advances the technical capabilities for thermal tolerance assessment in fishes but also establishes a foundation for future genetic and mechanistic studies aimed at understanding the molecular basis of thermal adaptation in aquatic ectotherms.

## Results

### Establishment of an automated temperature tolerance evaluation system

**Fig. 1:**
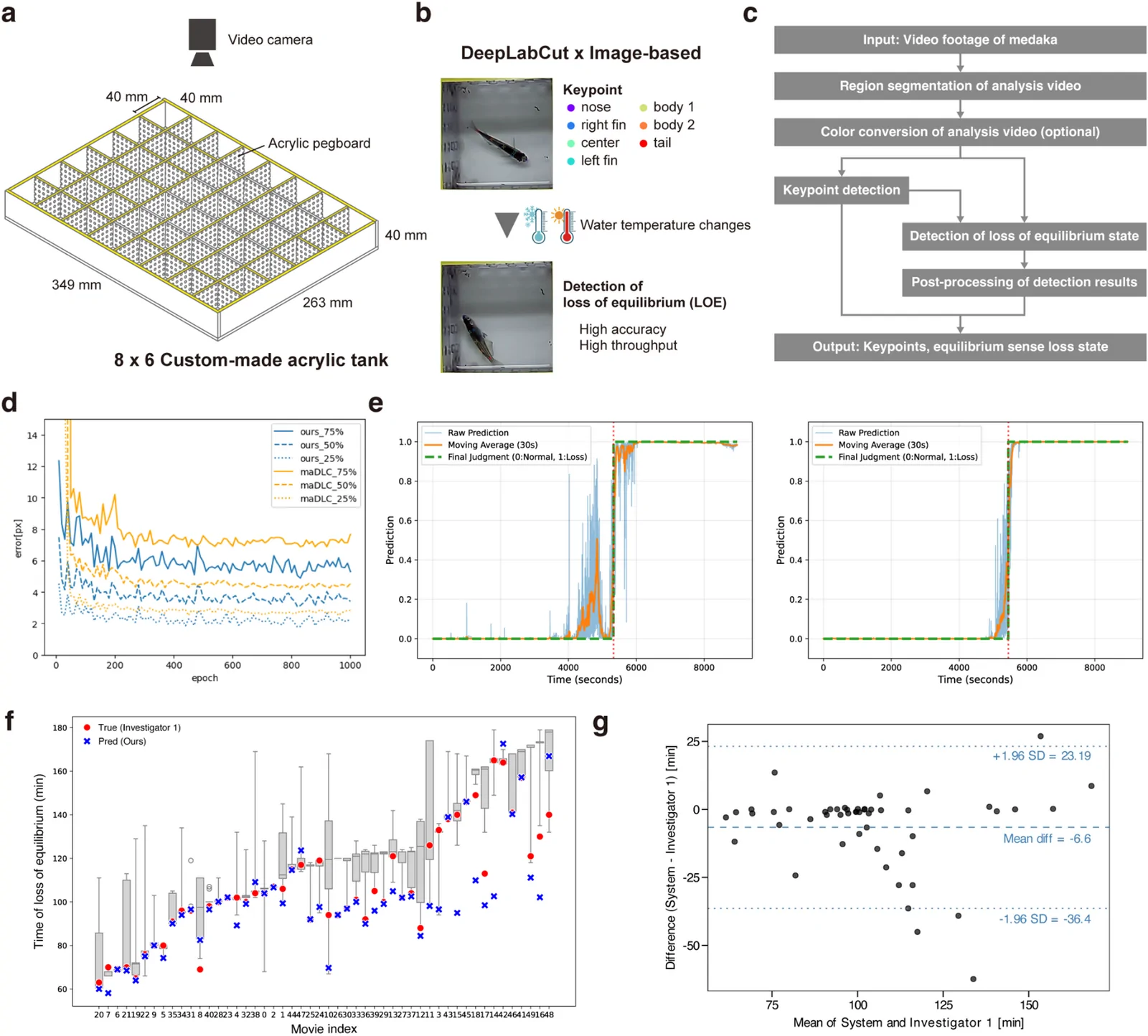
Automated temperature tolerance evaluation system for fish based on DeepLabCut. (**a**) Schematic illustration of the experimental setup. A custom-made acrylic tank (349 × 263 mm) with 48 compartments (40 × 40 mm each), separated by an acrylic pegboard, was placed in an incubator. Fish behavior was recorded from above using a video camera. (**b**) Overview of the DeepLabCut-based pose estimation and loss of equilibrium (LOE) detection. Seven anatomical keypoints (nose, right fin, center, left fin, body 1, body 2, and tail) were tracked in each frame. Upper image shows a fish swimming normally before temperature change; lower image shows a fish after LOE. (**c**) Flowchart of the automated analysis pipeline, from video input through region segmentation, color conversion, keypoint detection, LOE state classification, and post-processing to final output. (**d**) Comparison of keypoint detection accuracy between our region-splitting single-animal DLC approach (ours) and the multi-animal DLC baseline (maDLC) over 1,000 training epochs. The y-axis shows the Euclidean distance error (pixels) between predicted and annotated keypoint coordinates, and lines represent the 25th, 50th, and 75th percentiles of error across all keypoints evaluated on a held-out validation set of 30 frames. Lower values indicate higher accuracy. (**e**) Representative examples of automated LOE detection for two individual fish. Raw frame-by-frame predictions (light blue), 30-second moving average (orange), and final LOE judgment (green dashed line) are shown. The red dashed vertical line indicates the LOE time point determined by the system. (**f**) Validation of automated LOE detection against expert annotation. Box plots show the distribution of scores from the 12 naive annotators for each individual. Red circles indicate Investigator 1’s annotation; blue crosses indicate automated system predictions. (**g**) Bland-Altman plot comparing LOE timing between the automated system and Investigator 1 for the same 50 individuals shown in f. The x-axis shows the mean of the system and Investigator 1 values; the y-axis shows their difference (System − Investigator 1). The dashed line indicates the mean bias (− 6.6 min); dotted lines indicate the 95% limits of agreement (mean ± 1.96 SD; −36.4 to 23.19 min).

To establish an automated temperature tolerance evaluation system, we first developed an image processing pipeline based on DeepLabCut (Fig. 1a-c). While DeepLabCut offers powerful tracking capabilities, it faces limitations when handling multiple fish in partitioned tanks due to environmental variations and individual differences. To overcome these challenges, we implemented two key improvements: region partitioning based on physical tank divisions and a series of color transformations including shadow removal, color removal, and contrast enhancement (Fig. 1c). For the keypoint detection model, the seven anatomical keypoints (nose, left/right pectoral fins, center, anterior/posterior body, and tail) were manually annotated in selected frames. These annotated frames were processed through region partitioning and color transformation to generate 1,560 training images, with 174 images reserved for testing. To evaluate the effectiveness of these modifications, we compared keypoint detection accuracy between our region-splitting approach and standard multi-animal DeepLabCut (maDLC) over 1,000 training epochs, using the Euclidean distance error between predicted and annotated keypoint coordinates as the metric. Across all percentiles (25th, 50th, and 75th) of the error distribution, our approach consistently outperformed maDLC throughout training (Fig. 1d).

Next, we developed a method to accurately detect the timing of loss of equilibrium (LOE) by incorporating video frame information alongside the detected keypoints. LOE was operationally defined as the moment at which one of the two previously visible pectoral fins disappeared from view, indicating that the fish had begun to roll to one side and had lost postural equilibrium. For the LOE classification model, the behavioral state of each fish (0: normal, 1: LOE) was manually annotated at 3-second intervals from the recorded videos, independently of the keypoint annotation procedure. Our approach involved frame-by-frame classification of LOE state using a ResNet34-based model. The final fully connected layer was replaced with a linear layer projecting the 512-dimensional global average pooling output to a 64-dimensional feature vector. This frame feature was concatenated with the detected keypoint coordinates ([x, y] × 7 points) and their confidence scores (7 values), yielding an 85-dimensional combined representation (64 from frame features and 21 from keypoint data), which was passed through fully connected layers to produce a LOE probability score on a 0–1 scale. Classification results were converted into time-series data with a 30-second moving average and subsequent post-processing. Representative examples demonstrate that the system reliably identifies the transition from normal swimming to LOE state (Fig. 1e). This comprehensive analysis pipeline enables objective, observer-independent determination of LOE timing, providing a robust and efficient method for evaluating temperature tolerance in fish.

To validate the accuracy of the automated LOE detection system, we compared its output against manual annotations for 50 individuals. As the reference annotation, we used LOE timings scored by Investigator 1, who conducted the behavioral assays and was independent of the model training process. For comparison, LOE timings for the same videos were also independently scored by 12 naive annotators without prior experience in this assay. The automated system showed close agreement with Investigator 1’s annotation (Fig. 1f), with a mean absolute error (MAE) of 9.1 min, a root mean square error (RMSE) of 16.4 min, and a Spearman correlation of 0.85. Bland-Altman analysis revealed a mean bias of − 6.6 min, with 95% limits of agreement ranging from − 36.4 to 23.2 min, indicating a tendency for the system to detect LOE slightly earlier than Investigator 1 (Fig. 1g). Notably, the discrepancy between Investigator 1 and the median of naive annotators (MAE = 11.3 min, RMSE = 18.4 min) was larger than that between Investigator 1 and the automated system (MAE = 9.1 min, RMSE = 16.4 min) (Fig. 1f), indicating that the automated system performs at least as well as, if not better than, naive human observers in approximating expert-level judgment. These results demonstrate that our automated pipeline provides an objective and reproducible alternative to conventional manual LOE scoring.

To further contextualize the accuracy of the automated system, we compared LOE annotations between Investigator 1 and Investigator 2, both experienced with the experimental protocol and LOE criteria. The discrepancy between these two investigators (MAE = 9.5 min, RMSE = 18.0 min) was comparable to, or even slightly larger than, the discrepancy between the automated system and Investigator 1 (MAE = 9.1 min, RMSE = 16.4 min), indicating that the error of the automated system falls within the range of natural inter-observer variability among trained researchers. Moreover, while manual scoring is inherently subject to inter-observer variability, the automated system produces deterministic and fully reproducible outputs for a given video, eliminating this source of variability entirely. Therefore, in addition to achieving an accuracy comparable to inter-expert variability, the automated system offers the substantial advantage of consistent, observer-independent quantification, which is particularly valuable for large-scale or longitudinal studies where manual annotation by multiple observers may not be feasible.

### Medaka strains have different temperature tolerances

**Fig. 2:**
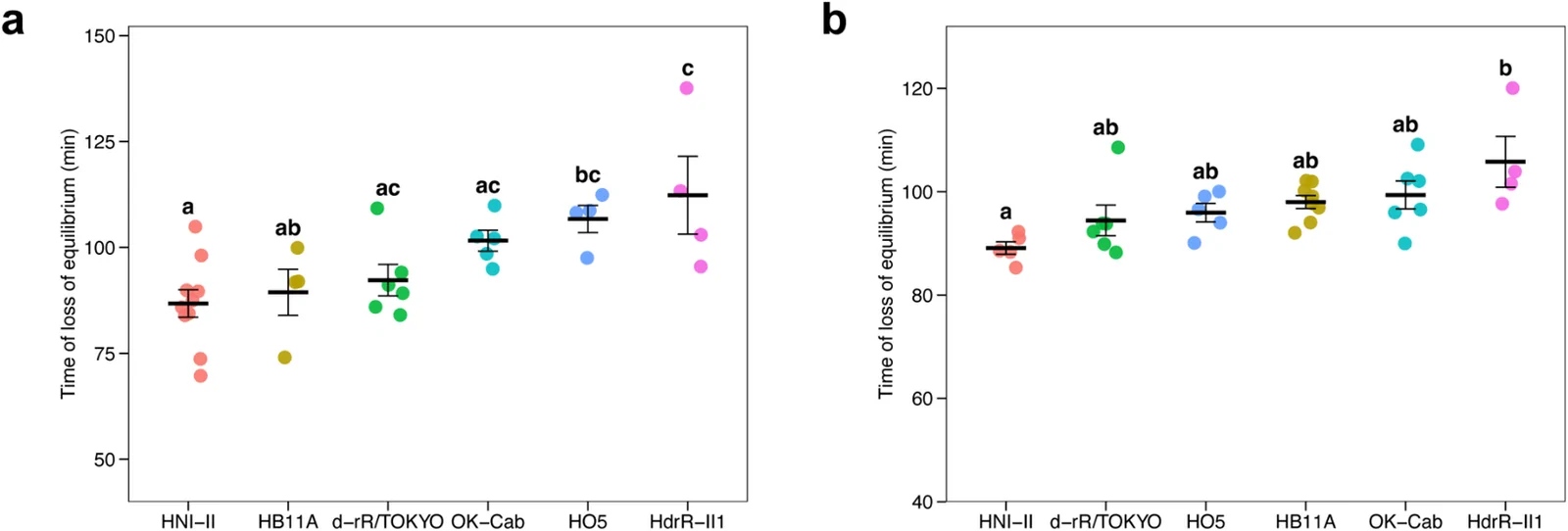
Temperature tolerance of medaka strains. (**a**) Cold tolerance of six medaka strains. LOE time was measured after rapidly decreasing the incubator temperature from 26 to 2 °C. Sample sizes were as follows: HNI-II (n = 10), HB11A (n = 4), d-rR/TOKYO (n = 6), OK-Cab (n = 5), HO5 (n = 4), and HdrR-II1 (n = 4). (**b**) Heat tolerance of six medaka strains. LOE time was measured after rapidly increasing the incubator temperature from 26 °C to 55 °C. Sample sizes were as follows: HNI-II (n = 5), d-rR/TOKYO (n = 6), HO5 (n = 5), HB11A (n = 8), OK-Cab (n = 6) and HdrR-II1 (n = 6). Data are presented as mean ± SEM. Each dot represents an individual fish. Groups sharing the same letter are not significantly different (adjusted *p* value > 0.05), based on one-way ANOVA followed by Tukey’s post hoc test.

Since various strains have been established in medaka, we next evaluated the temperature tolerance of three *O. latipes* inbred strains (HdrR-II1, HO5 and HB11A), one *O. sakaizumii* inbred strain (HNI-II), and two laboratory strains (d-rR/TOKYO and OK-Cab) using our automated quantification system. Initially, adult medaka acclimated at 26 °C were transferred to tanks placed in an incubator. The temperature was rapidly decreased by setting the incubator temperature to 2 °C, and the fish behavior was recorded for several hours. Analysis of the recorded videos using our automated system revealed strain-specific differences in the timing of LOE. HNI-II, HB11A, d-rR/TOKYO, OK-Cab, HO5 and HdrR-II1 strains lost equilibrium at 86.8 ± 3.3, 89.4 ± 5.5, 92.3 ± 3.7, 101.6 ± 2.5, 106.7 ± 3.2 and 112.3 ± 9.2 min (mean ± SEM) after temperature reduction, respectively (Fig. 2a). One-way ANOVA followed by Tukey’s post hoc test revealed a gradual pattern of overlapping differences in LOE timing among the strains (Fig. 2a). HdrR-II1 showed the longest mean LOE time and differed significantly from HNI-II (adjusted *p* value < 0.01) and HB11A (adjusted *p* value < 0.05), while sharing overlapping statistical grouping with d-rR/TOKYO, OK-Cab, and HO5, indicating that HdrR-II1 was among the most cold-tolerant strains tested rather than uniquely superior. HNI-II showed the shortest mean LOE time and differed significantly from HdrR-II1 and HO5.

Next, we evaluated high-temperature tolerance in the same strains as in the cold tolerance assay by rapidly increasing the incubator temperature from 26  to 55 °C. The automated analysis revealed that HNI-II, d-rR/TOKYO, HO5, HB11A, OK-Cab, and HdrR-II1 strains lost equilibrium at 89.1 ± 1.2, 94.4 ± 3.0, 96.0 ± 1.8, 98.0 ± 1.3, 99.4 ± 2.7, and 105.8 ± 4.9 min (mean ± SEM) after temperature elevation, respectively (Fig. 2b). One-way ANOVA followed by Tukey’s post hoc test revealed a significant difference only between HdrR-II1 and HNI-II (adjusted *p* value < 0.01), indicating that HdrR-II1 exhibited the longest mean LOE time and was among the most heat-tolerant strains tested, whereas HNI-II showed the lowest heat tolerance. No other pairwise comparisons reached statistical significance. These results indicate that HNI-II showed comparatively low tolerance to both cold and heat stress among the tested strains, whereas the relative ranking of the other strains showed some variation between cold and heat stress conditions, consistent with the largely overlapping statistical groupings observed in both assays.

### Cold tolerance varies markedly among *Oryzias *species

**Fig. 3:**
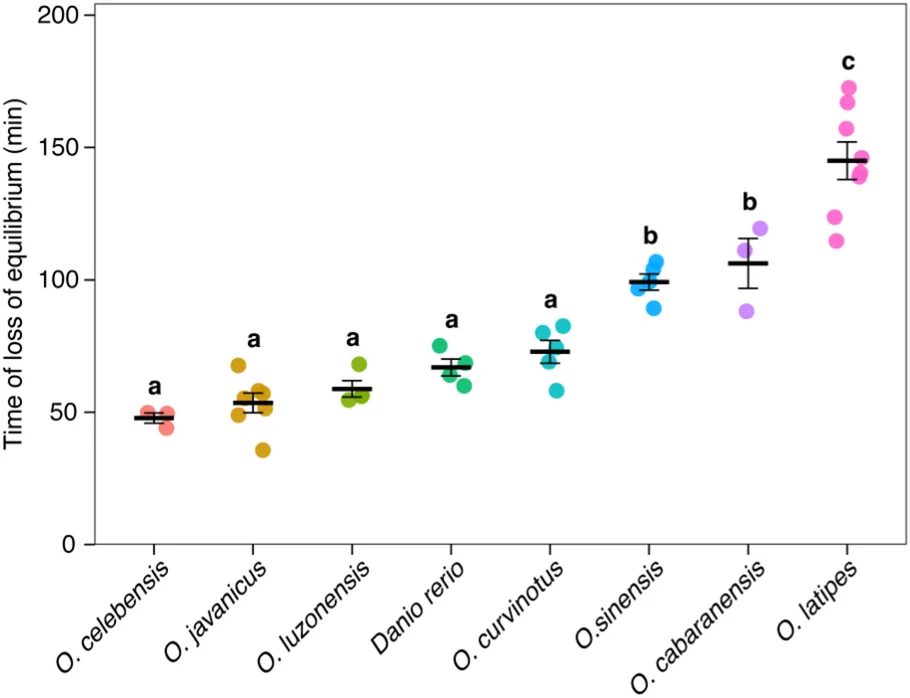
Cold tolerance of *Oryzias* species and zebrafish. LOE time was measured after rapidly decreasing the incubator temperature from 26 to 2 °C. Sample sizes were as follows: *O. celebensis* (n = 3), *O. javanicus* (n = 7), *O. luzonensis* (n = 4), *Danio rerio* (n = 4), *O. curvinotus* (n = 5), *O. sinensis* (n = 5), *O. cabaranensis* (n = 3), and *O. latipes* (n = 8). Data are presented as mean ± SEM. Each dot represents an individual fish. Groups sharing the same letter are not significantly different (adjusted *p* value > 0.05), based on one-way ANOVA followed by Tukey’s post hoc test.

To further explore the diversity of cold tolerance across the genus *Oryzias*, we evaluated the cold tolerance of medaka (*O. latipes*) and six medaka-related species (*O. sinensis*, *O. cabaranensis*, *O. curvinotus*, *O. luzonensis*, *O. celebensis* and *O. javanicus*) along with zebrafish (*Danio rerio*) as a representative model fish species. For *O. latipes*, we used a genetically uncontrolled, outbred wild-type population rather than a single inbred or laboratory strain, because our results (Fig. 2) demonstrates that thermal tolerance can vary considerably among medaka strains, and we considered an outbred population to be a more representative sample of the species as a whole for the purpose of this interspecific comparison. One-way ANOVA followed by Tukey’s post hoc test revealed substantial interspecific variation in cold tolerance (Fig. 3). *O. latipes* exhibited the highest cold tolerance among the tested species, with a mean LOE time of 145.0 ± 7.1 min, which was significantly longer than all other species. *O. cabaranensis* (106.0 ± 9.4 min) and *O. sinensis* (99.2 ± 3.0 min) formed a group of relatively high cold tolerance, showing no significant difference from each other but significantly higher tolerance than *O. curvinotus*, *Danio rerio*, *O. luzonensis*, *O. javanicus* and *O. celebensis*. The remaining five species—*O. curvinotus* (72.8 ± 4.4 min), *Danio rerio* (66.9 ± 3.2 min), *O. luzonensis* (58.8 ± 3.1 min), *O. javanicus* (53.5 ± 3.7 min), and *O. celebensis* (47.7 ± 1.9 min)—did not differ significantly from one another, indicating a broadly overlapping range of lower cold tolerance among these species. These results demonstrate that cold tolerance varies markedly among *Oryzias* species, broadly reflecting the latitudinal distribution of their native habitats.

## Discussion

In this study, we developed a DeepLabCut-based automated system for quantifying the timing of LOE during thermal stress assays in fish. Using this system, we revealed distinct differences in temperature tolerance among medaka strains and related *Oryzias* species, demonstrating that genetic background influences temperature tolerance capabilities. Cold tolerance varied among the tested medaka strains, and interspecific comparisons across the genus *Oryzias* revealed substantial variation in cold tolerance that broadly corresponded with the latitudinal distribution of native habitats. Taken together, our automated system provides a robust platform for high-throughput temperature tolerance evaluation in fish, opening new possibilities for investigating the genetic and physiological mechanisms underlying temperature adaptation.

Conventional methods for evaluating temperature tolerance in fish have relied heavily on manual observation to detect LOE, requiring continuous monitoring over extended periods. This approach not only demands significant time and labor but also introduces potential inconsistencies due to observer subjectivity and fatigue during long observation periods. To assess whether our automated system overcomes these limitations, we validated its accuracy against manual annotation. The automated system achieved an accuracy comparable to the natural variability observed between two trained investigators (MAE = 9.1 min, RMSE = 16.4 min for system vs. Investigator 1, compared to MAE = 9.5 min, RMSE = 18.0 min between Investigator 1 and Investigator 2), and outperformed naive human observers without prior experience in this assay (MAE = 11.3 min, RMSE = 18.4 min). Importantly, unlike manual scoring, which is inherently subject to inter-observer variability, the automated system produces deterministic and fully reproducible outputs for a given video. This combination of accuracy and reproducibility makes the system particularly advantageous for large-scale or longitudinal studies where manual annotation by multiple observers is not feasible. The automated system also showed a tendency to detect LOE slightly earlier than Investigator 1, with a mean bias of − 6.6 min (95% limits of agreement: −36.4 to 23.2 min; Fig. 1g). Because the classification model was trained on annotations provided by Investigator 2, this bias likely reflects subtle differences in LOE judgment criteria between the two investigators, rather than a systematic limitation of the automated approach itself. This observation illustrates that, even among trained researchers, the precise criteria used to judge LOE can vary subtly, and the output of the automated system inevitably reflects the annotation criteria used during training.

Our automated system revealed that medaka strains exhibit a gradual pattern of differences in temperature tolerance. Taking advantage of the numerous well-characterized strains available in medaka^15, 16^, previous studies have examined temperature tolerance at the embryonic and cellular levels. Hirayama et al.^17^ reported that cell lines derived from the northern Japanese lineage (*O. sakaizumii*) showed greater proliferation at low temperatures compared to those from the southern lineage (*O. latipes*), whereas southern-derived lines showed higher heat tolerance. Similarly, Yoshimura et al.^18^ reported that HNI-II embryos, derived from *O. sakaizumii*, maintained more stable cardiac rhythm under low-temperature conditions than HdrR-II1, an *O. latipes*-derived strain. Notably, our adult behavioral assay revealed a pattern that contrasts with these prior findings. HNI-II showed the lowest mean tolerance to both cold and heat stress among the tested strains, whereas HdrR-II1 showed among the longest LOE times under cold stress. These discrepancies likely reflect fundamental differences in the mechanisms governing temperature tolerance across life stages. In embryos and cell lines, temperature tolerance is largely determined by cell-autonomous properties such as membrane fluidity and protein stability, whereas LOE in adult fish represents an integrative physiological response involving the nervous system, musculature, and cardiovascular system. These findings suggest that the relationship between geographical origin and temperature tolerance is more complex at the whole-organism level than previously assumed from embryonic or cellular studies. It should also be noted that the present strain panel—selected based on availability through NBRP Medaka—includes only one *O. sakaizumii*-derived strain (HNI-II), alongside three *O. latipes* inbred strains (HdrR-II1, HO5, HB11A) and two additional *O. latipes* laboratory strains (d-rR/TOKYO, OK-Cab) that are relatively closely related to one another. This limits the extent to which the present dataset can be taken as representative of thermal tolerance diversity across medaka strains as a whole. Future studies incorporating a broader panel of genetically diverse strains, including additional northern-derived lines, will be needed to draw more general conclusions.

Another notable advantage of the medaka system is the existence of numerous closely related species inhabiting diverse thermal environments. Our automated system revealed substantial differences in cold tolerance among *Oryzias* species. *O. latipes* exhibited by far the longest LOE time, followed by *O. cabaranensis* (a species endemic to northern Taiwan that was recently described as distinct from *O. sinensis*;^19^), *O. sinensis*, *O. curvinotus*, *Danio rerio*, *O. luzonensis*, *O. javanicus* and *O. celebensis*, broadly corresponding with the latitudinal distribution of their native habitats. Notably, both *O. cabaranensis* and *O. sinensis* showed relatively high cold tolerance despite their lower-latitude distributions, suggesting that factors beyond latitude alone may also contribute to cold tolerance. These results support the general relationship between geographical distribution and cold tolerance, while highlighting that cold tolerance evolution within the genus *Oryzias* may be shaped by a complex interplay of phylogenetic history, latitude, and local environmental conditions.

In this study, we successfully automated the detection of LOE, which has traditionally relied on subjective visual observation. Although we employed medaka, our system is built upon the DeepLabCut framework and can potentially be adapted to other fish species and animal taxa. Furthermore, medaka strains and their related species offer unique advantages for dissecting the genetic basis of temperature tolerance: inter-strain hybridization is feasible, enabling forward genetic approaches, and biological resources including live strains and genomic data are readily accessible through established bioresource centers such as NBRP Medaka and MedakaBase^20^. Comparative analyses exploiting the diversity in temperature tolerance revealed in this study, combined with the rich genetic and genomic toolkit available in the medaka system, are expected to advance our understanding of the molecular mechanisms underlying temperature adaptation in fish.

## Materials and methods

### Animals

Medaka (*Oryzias latipes*) and zebrafish (*Danio rerio*) were obtained from a local dealer (Fuji 3 A Project, Nagoya, Japan). These fish represented outbred wild-type stocks and were not assigned to any specific inbred or laboratory strain. They were used for interspecific comparisons in Fig. 3. Medaka strains derived from the southern Japanese lineage (*Oryzias latipes*; HdrR-II1, HO5 and HB11A) and the northern Japanese lineage (*Oryzias sakaizumii*; HNI-II), as well as two laboratory strains (d-rR/TOKYO and OK-Cab) and related species (*O. sinensis*, *O. cabaranensis*, *O. curvinotus*, *O. luzonensis*, *O. celebensis* and *O. javanicus*) were supplied by NBRP Medaka (https://shigen.nig.ac.jp/medaka/). All medaka and zebrafish were hatched from eggs and reared under long-day (14 h light / 10 h dark; 26 °C) conditions in a housing system (MEITO system, Meito Suien) until adulthood. All experiments in this study were performed using sexually mature medaka and zebrafish (at least 2 months old). All animal studies were carried out in accordance with the ARRIVE guidelines. All methods complied with the relevant guidelines and regulations and were approved by the Animal Experiment Committee of Nagoya University.

### Developing automated temperature tolerance evaluation system

DeepLabCut was employed as the foundation of our automated temperature tolerance evaluation system. We configured it to track seven anatomical keypoints of medaka (nose, left/right pectoral fins, center, anterior/posterior body, and tail) in video recordings. For keypoint detection improvement, we implemented two preprocessing steps. First, we developed a region partitioning algorithm that automatically detects and segments individual fish chambers using HSV color thresholding ((h, s,v) values of (20,50,40) to (60,255,255)) and contour analysis. Second, we optionally applied three color transformations depending on video recording conditions: shadow removal using background subtraction and gamma correction, color removal through HSV conversion with saturation elimination, and contrast enhancement using an S-curve tone mapping. For the keypoint detection model, the seven anatomical keypoints were manually annotated in selected frames. These manually annotated frames were processed through region partitioning and color transformation to generate 1,560 training images, with 174 images reserved for testing. A ResNet50 backbone with Group Normalization, pretrained on ImageNet, was used for keypoint detection. The model was trained for 300 epochs using AdamW optimizer (initial learning rate = 0.0005) with a step-wise learning rate schedule (reduced to 0.0001 at epoch 90 and 1 × 10^− 5^ at epoch 120). Data augmentation included affine transformations (rotation ± 30°, scaling 0.5–1.25×), motion blur, and Gaussian noise, with input images cropped to 448 × 448 pixels.

To detect LOE, a classification model was developed by combining both key point data and video frame information. For the purpose of manual annotation, LOE was operationally defined as the moment at which one of the two previously visible pectoral fins disappeared from view, indicating that the fish had begun to roll to one side and had lost postural equilibrium. The model architecture consisted of ResNet34 for frame feature extraction, in which the final fully connected layer was replaced with a linear layer projecting the 512-dimensional global average pooling output to a 64-dimensional feature vector. This frame feature vector was then concatenated with the detected keypoint coordinates ([x, y] × 7 points) and their confidence scores (7 values), yielding an 85-dimensional combined representation. This was subsequently passed through fully connected layers (Linear(85→32), followed by Linear(32→1) with a Sigmoid activation) to produce the LOE probability score. For the LOE classification model, the behavioral state of each fish (0: normal, 1: LOE) was manually annotated at 3-second intervals from the recorded videos, independently of the keypoint annotation procedure. Data augmentation including random horizontal/vertical flips and color jittering was applied. The model was trained using binary cross-entropy loss with Adam optimizer (learning rate = 0.0001) for 10 epochs with an input size of 224 × 224 pixels. For post-processing, we implemented a three-step pipeline: (1) conversion of frame-wise predictions to time-series data, (2) application of a 30-second centered moving average, and (3) thresholding at 0.5 followed by rule-based smoothing. The time point of transition from 0 to 1 in the processed data was identified as the LOE timing.

### Behavioral tests

A custom-made acrylic tank (W349 × D263 × H40 mm) with 48 compartments (40 mm × 40 mm each) was placed in an incubator (MIR-154-PJ, Panasonic or MIR-153, SANYO) and filled with 1 L of fish tank water. Before the experiment, individual fish were introduced into each compartment and acclimated at 26 °C for 1 h. After 1 h, temperature stress was induced by rapidly changing the incubator temperature from 26 °C to 2 °C or 26 °C to 55 °C. Fish behavior was recorded for several hours using a camera (ELP-USB4KCAM01H-CFV (2.8–12), Ailipu Technology Co., Ltd) mounted at the top of the incubator. The recorded videos were analyzed using our automated temperature tolerance evaluation system to determine the timing of LOE for each individual fish.

Water temperature within the experimental chambers was recorded at 10-minute intervals throughout both the cold tolerance and heat tolerance assays (*n* = 4 tests for each condition) using a data logger (TR42A, T&D Corporation, Matsumoto, Japan) equipped with a waterproof temperature sensor (TR-5106, T&D Corporation) (Supplementary Fig. 1).

### Statistical analysis

All statistical analyses were performed using R (version 4.5.2). Data are presented as means ± SEM. For comparisons, one-way ANOVA followed by Tukey’s post hoc test was performed.

## Electronic Supplementary Material

Below is the link to the electronic supplementary material.

Supplementary Material 1

## Data Availability

All data are available from the authors upon request. All codes used in the analysis are available at https://github.com/nakayama-tomoya/dlc-loe-detector.
