# Supplementary material for: DeepLabCut-based automated system reveals diverse temperature tolerance among medaka strains and related Oryzias species
Source: Sci Rep. 2026 Sep 10;16:25893. doi: 10.1038/s41598-026-66712-w (PMC13562581; doi:10.1038/s41598-026-66712-w)
Supplement: Supplementary file 1 [file 41598_2026_66712_MOESM1_ESM.docx]

**Supplementary Figure 1. Water temperature profiles during the thermal tolerance assays.**

Mean water temperature (± SEM) measured within the experimental chambers during the cold tolerance assay (a) and heat tolerance assay (b), in which the incubator temperature was rapidly decreased from 26°C to 2°C (a) or increased from 26°C to 55°C (b). Water temperature was recorded at 10-minute intervals throughout each assay (n = 4 tests for each condition).
